# Can Increased Education to the Public Optimize Aging? Results of Structured Interviews with Older Experts in Aging

**DOI:** 10.1093/geroni/igaf122.2472

**Published:** 2025-12-31

**Authors:** Sue Hazelett, F Michael Martin

**Affiliations:** Summa Health System, Akron, Ohio, United States; Retired, Williamsburg, Virginia, United States

## Abstract

The first major cohort of experts in aging who developed the theories that guide today’s best practices in geriatrics and gerontology are now experiencing aging themselves. We examined strategies used by retired aging-related experts throughout their own aging experience to determine whether the best practices that guided their professional practice impacted and are validated by their own lived aging experience. A convenience sample of experts age > 65 in the fields of gerontology, geriatrics, and/or aging were interviewed using a structured survey. Questions focused on their aging experience and how their career-associated expertise affected it. Nineteen interviews were completed with an average age of 75.4, 63% male, and 21% living alone. 16 of 19 (84%) indicated they have experienced physical or mental health issues and yet 95% consider themselves aging well. Factors rated by respondents as important contributors to this were maintaining purpose (89%), keeping/ forming relationships (100%), maintaining health (84%), coping with losses (100%), and preparing for functional decline (95%). They reported their professional knowledge allowed them to better prepare for inevitable losses through intentional physical activity, maintaining personal relationships, financial planning, and better lifestyle choices. 84% rated their previous career as important in helping them age successfully. These results suggest that greater efforts towards widespread education of community-based adults regarding common aging-related issues prior to them reaching retirement age could significantly improve the aging experience for large portions of the population by allowing them to prepare for likely age-related outcomes, just as these aging experts found for themselves.

